# Clinical Efficacy of Prolotherapy for Temporomandibular Joint Disorders: A Systematic Review and Meta-Analysis

**DOI:** 10.3390/clinpract15030051

**Published:** 2025-02-27

**Authors:** Antonios Saramantos, Athanassios Kyrgidis, Gregorios Venetis, Georgios Hatziantoniou, Anestis Chrysostomidis, Chrysanthi Sardeli, Ioannis Tilaveridis

**Affiliations:** 1 Department of Oral & Maxillofacial Surgery, Aristotle University of Thessaloniki, Specialized Cancer Treatment and Reconstruction Center, General Hospital of Thessaloniki “George Papanikolaou”, 57010 Thessaloniki, Greece; saramantosant@gmail.com (A.S.); ghatziadoniou@gmail.com (G.H.); a.chrisostomidis@gmail.com (A.C.); jtilaver@yahoo.com (I.T.); 2 Laboratory of Oral & Maxillofacial Surgery, Dental School, Aristotle University of Thessaloniki, 54124 Thessaloniki, Greece; gvenetis@dent.auth.gr; 3 Laboratory of Clinical Pharmacology, Medical School, Aristotle University of Thessaloniki, 54124 Thessaloniki, Greece; sardeli@auth.gr

**Keywords:** temporomandibular joint, temporomandibular disorder, prolotherapy, placebo, systematic review, meta-analysis, randomized controlled trials

## Abstract

**Background:** Temporomandibular disorders (TMDs) encompass a group of conditions characterized by anatomical, histological, and/or functional abnormalities that affect the muscular and/or articular components of the temporomandibular joint. Prolotherapy is an injectable treatment modality for chronic musculoskeletal pain that involves dextrose solution administration in the joint. **Aims:** To summarize, the aims involve considering the existing quality of clinical evidence on the efficacy of prolotherapy versus placebo and other active comparators, such as autologous blood products or botulinum toxin, in improving the outcomes of TMDs. **Methods:** A literature search in MEDLINE, Scopus, and Cochrane databases was performed, following the PRISMA statement guidelines, to identify randomized controlled trials (RCTs) of patients with TMDs receiving prolotherapy. The maximal incisor opening (MIO), visual analogue score (VAS) for pain, and frequency of dislocations were analyzed as the outcomes. The weighted mean difference was used to pool outcomes. The risk of bias was recorded for the included studies. **Results:** Six studies comparing prolotherapy to placebo were identified. Prolotherapy is uniformly more efficient in reducing the VAS for pain when compared to the placebo (mean difference = 1.20, 95%CI: 0.56–1.84, *p* < 0.001). Perceived jaw mobility was improved among prolotherapy patients, (mean difference = 0.47, 95%CI: 0.05–0.90, *p* = 0.003) when compared to the placebo. A beneficial effect for prolotherapy with regard to MIO (mean difference = 0.84, 95%CI: −2.12–3.80, *p* = 0.58) was not confirmed. Prolotherapy appears to be more efficient than autologous blood products in reducing VAS for pain (mean difference = 0.49, 95%CI: 0.11–0.87, *p* = 0.01). Prolotherapy was found to be more effective in reducing pain, MIO, and clicking when compared to an occlusal splint in a single study. **Conclusions:** Prolotherapy is also a promising modality for TMDs, despite the limited number of randomized clinical trials. Existing evidence supports its use to reduce TMD-related pain, even against other modalities. Further research is needed to better describe the benefit of prolotherapy for other outcomes.

## 1. Introduction

Temporomandibular disorders (TMDs) encompass a group of conditions characterized by anatomical, histological, and/or functional abnormalities affecting the muscular and/or articular components of the temporomandibular joint (TMJ) [1]. These disorders are marked by pain confined but not limited to the TMJ or surrounding tissues and functional limitations in jaw mobility, like difficulty chewing, quick jaw fatigue, nocturnal teeth grinding (bruxism), the feeling of tension in the jaw, or clicking sounds during jaw movements. Females aged 20 to 40 years have been reported to be the most affected patient group [1]. Estrogen levels are thought to be associated with pain modulation in the temporomandibular joint, although this effect has not been confirmed in systematic reviews [2]. The global prevalence of TMDs ranges between 31% [3] and 34% [4]. The annual incidence of painful TMDs is estimated to be between 3 and 12% in various studies [1,5,6,7,8]. While the natural progression of TMDs is not well documented, studies report recurrence in 65% and chronic symptoms in 19% of affected individuals. The condition is characterized by periods of automatic remission, followed by relapses, with variable intervals [5,6]. Clinical research for TMDs is further complicated by their differential causality and lack of use of common diagnostic criteria by the whole research community [8,9]. To address this, the Research Diagnostic Criteria for TMDs (RDC/TMD) were proposed some years ago [9] which are a classification of TMDs by their probable etiology. The extent to which RDC/TMD classification informs guideline-based care remains to be better elucidated [5,9].

Nowadays, non-surgical therapeutic approaches are considered the treatment of choice, unlike in previous decades [1,5]. Practical recommendations comprise patient education, self-care strategies, simple analgesics, dental occlusal splints, physiotherapy, and even acupuncture. However, systematic reviews have yet to identify a universally superior treatment modality [5]. Evidence supporting injection therapies for TMDs remains limited. Existing treatment modalities comprise intra-articular corticosteroids or hyaluronic acid injections for inflammatory TMDs or TMJ osteoarthritis, respectively, and the intramuscular botulinum toxin for TMJ-related myofascial pain and simple arthrocentesis—lavage using normal saline [10]. Nonetheless, many studies on these therapies suffer from small sample sizes, short durations, methodological inconsistencies, and variable outcomes, making it difficult to draw definitive clinical recommendations [1,5,6]. Given the limitations of all various existing treatments mentioned immediately above, rigorous clinical research to explore novel modalities is of value.

Hypertonic dextrose prolotherapy (HDPT) is an injectable treatment modality for chronic musculoskeletal pain comprising intra-articular dextrose solution administration [11,12,13]. While the pathophysiology for its efficacy is not completely determined, it is traditionally thought to promote healing and pain relief by triggering a transitory inflammatory response, leading to increased cell proliferation. Emerging research suggests that HDPT’s effects may be multifactorial, including direct sensorineural impacts. Recently, higher-quality clinical trials have examined HDPT’s efficacy in TMD management, reporting favourable outcomes on pain reduction and functional improvement using standardized measures. However, these findings were not included in previous systematic reviews. An ongoing evaluation of evolving medical evidence is critical to guide clinical decision-making and improve patient care.

It is common knowledge that randomized controlled trials are the superior form of medical evidence upon which to build appropriate clinical conclusions [14].

Thus, the aim of the present study was to determine whether prolotherapy could be a useful modality addition to the variety of existing treatments. For that, we attempted to systematically summarize currently available evidence originating from randomized controlled trials (RCTs) that are evaluating HDPT’s effectiveness in managing patients with TMDs, examining all available outcomes.

## 2. Materials and Methods

### 2.1. Guidelines Followed

**Figure 1:**
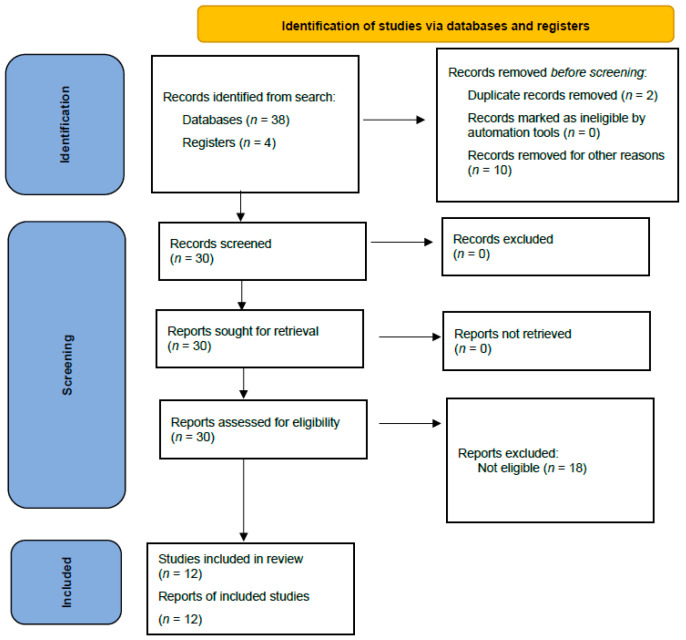
Flowchart of the systematic review.

This systematic review followed the guidelines outlined in the PRISMA statement [15]. Review protocol preregistration was not performed. Figure 1 displays the flow chart diagram.

### 2.2. Search Strategy

To identify eligible studies, two independent investigators (A.S, A.K) conducted a thorough literature search in the following electronic databases: MEDLINE (PubMed), Scopus, and Cochrane (CENTRAL), from the year 1990 to September 2024 and arising discrepancies were resolved by a third investigator (G.H). Scopus was used to avoid excluding studies not indexed in MEDLINE and CENTRAL because it only includes RCTs and features a rigorous handsearching process. In addition, conferences and grey literature were screened, and a manual search of references of included studies was conducted to search for relevant studies. A representative example of a search string in PubMed is: ((“Tempormandibular” [MeSH Terms] OR “TMJ” [Title/Abstract] OR “Temporomandibular joint” [Title/Abstract] OR “temporo*” [Title/Abstract] OR “mandibular*” [Title/Abstract] OR “joint hypermobility” [Title/Abstract] OR “subluxation” [Title/Abstract] AND (“prolotherapy” [MeSH Terms] OR “dextrose*” [Title/Abstract] OR “prolo*” [Title/Abstract] OR “dextrose prolotherapy “ [Title/Abstract] OR “prolo*” [Title/Abstract]. Database search queries are presented in Supplementary Table S1.

### 2.3. Inclusion Criteria

The following parameters were set as inclusion criteria: Randomized controlled clinical studies that included patients with TMDs, in whom prolotherapy had to be administered to at least one trial arm. The injection protocol ought to include an intra-articular injection, complemented or not by additional injections to the periarticular soft tissues. Comparator arms would be placebo (i.e., normal saline injections), active comparators (i.e., blood injections) or other types of interventions (i.e., physiotherapy). Studies with multiple interventions were considered as long as they were uniform across all study groups such that the clean efficacy attributed to prolotherapy could be estimated. The PICO framework and inclusion criteria are presented in Supplementary Table S2.

### 2.4. Data Extraction

Two of the authors independently extracted data from studies that met the eligibility criteria. A standardized form was used to record the following parameters: (i) first author, (ii) year of publication, (iii) country of conduct, (iv) study design, (v) follow-up length, (vi) absolute number of patients participated in the study in each arm, (vii) treatment modalities applied to each arm, and (viii) outcomes reported in every study [i.e., maximum opening/visual analogue pain score].

### 2.5. Risk of Bias and Study Quality Assessment

The quality of the selected studies was assessed using the RevMan Risk of Bias tool. The risk of publication bias was examined with funnel plots, presented in the Supplementary Figures (Figures S1–S4).

### 2.6. Statistical Analysis

Post-intervention continuous outcomes in different study arms were pooled as the weighted mean difference (WMD) with 95% confidence intervals (CI). Both fixed and random effect models were used for data synthesis, in cases of high heterogeneity. The I^2^ index was preferred for determining heterogeneity extent among studies, with values lower than 35% being considered as low heterogeneity, values ranging from 35% to 65% as moderate and values > 65% as considerable heterogeneity. More conservative random effects models were used when heterogeneity was moderate or considerable. Attempts to calculate the standard deviation (SD) from the Inter Quartile Range (IQR) were made when data were not available. The transformation of outcomes from one scale to another scale was also attempted in a few studies. Subgroup analyses and sensitivity analyses were conducted to investigate possible sources of heterogeneity. The random effects model was used to address high heterogeneity. All statistical tests were two-sided, *p*-value < 0.05 was considered statistically significant, and the analysis was conducted with Review Manager v.5.4.1.

## 3. Results

### 3.1. Description of Results from Literature Search

**Figure 2:**
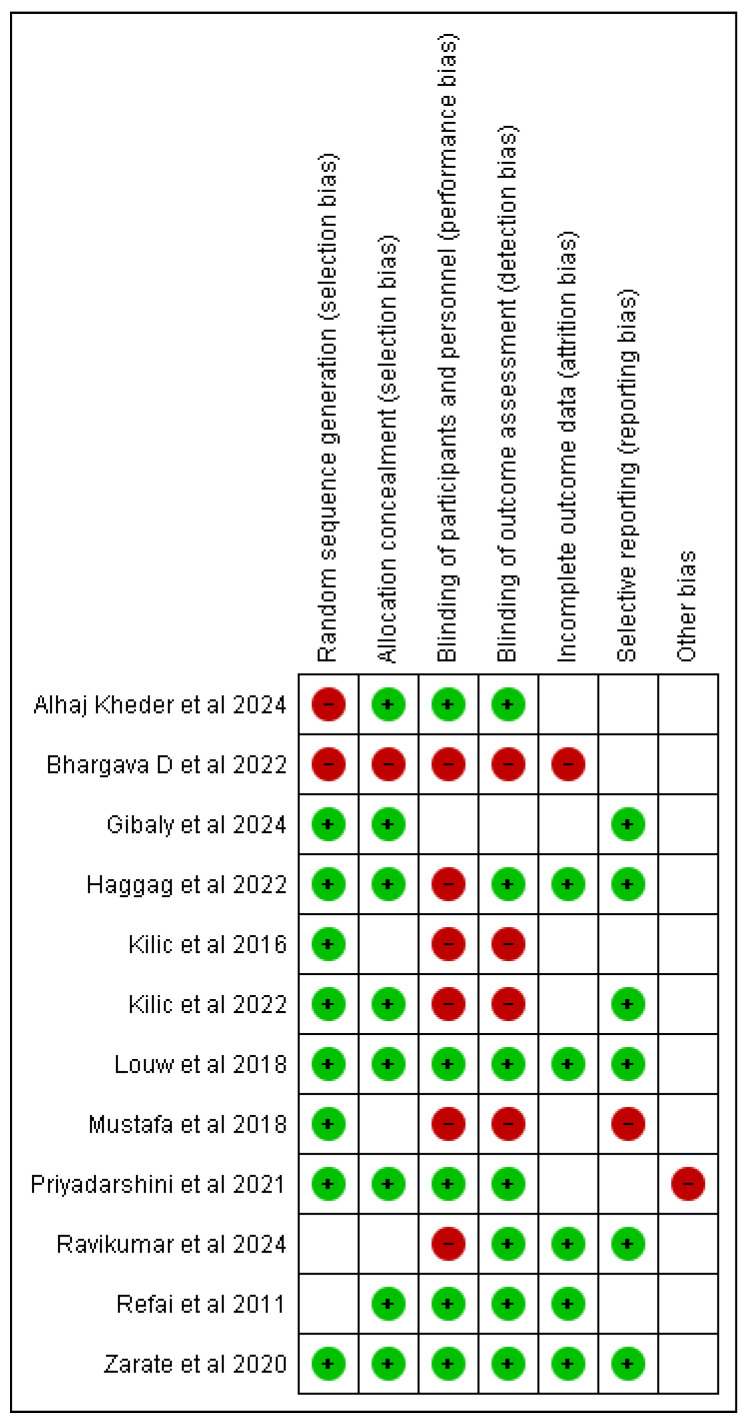
Risk of bias summary: review authors’ judgements about each risk of bias item for each included study [16,17,18,19,20,21,22,23,24,25,26,27]. Green: low risk; white: unclear risk; red: high risk of bias.

**Figure 3:**
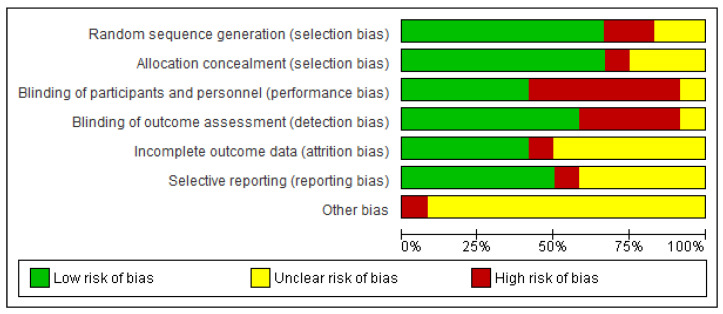
Risk of bias graph: review authors’ judgements about each risk of bias item presented as percentages across all included studies [16,17,18,19,20,21,22,23,24,25,26,27]. Green: low risk; Yellow: unclear risk; red: high risk of bias.

We identified 42 citations from all searches. After screening the titles and abstracts, we retrieved 30 full texts for further assessment. Of these, 18 were excluded for the following reasons: trial without a control arm (*n* = 10), review (*n* = 4), and articles not related to the topic (*n* = 4). Twelve full texts were included for descriptive synthesis (Table 1 and Table 2) [16,17,18,19,20,21,22,23,24,25,26,27], among which eight were included in quantitative synthesis [16,18,20,21,22,23,24,26] (Figure 1). The risk of bias assessment of the studies included in the quantitative syntheses is presented in Figure 2 and Figure 3.

### 3.2. Modalities Synthesized

**Figure 4:**
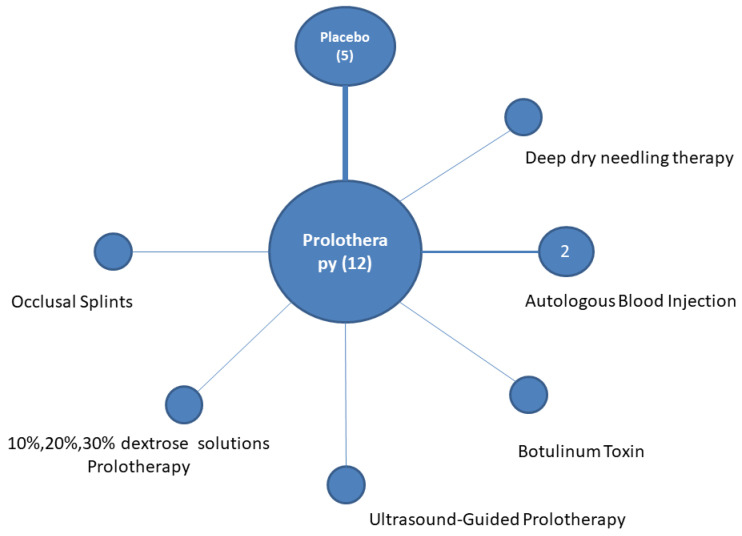
Network of studies [16,17,18,19,20,21,22,23,24,25,26,27] included in the systematic review.

We identified six studies comparing prolotherapy to placebo [16,20,21,22,23,24]. Two studies compared prolotherapy to autologous blood product injection [18,26]. One study examined a dry needling comparator as the placebo, while the remainder [20,21,22,23,24] used normal saline, commonly added to topical anesthetics such as lidocaine in the majority of cases and bupivacaine in a few cases [18]. Botulinium toxin was the active comparator in a single study [19], while dental occlusal splints were the active comparator in another one [27]. One study examined the adjunct of ultrasound (U/S) to assist the prolotherapy injection [17], while another study was designed to examine dextrose dose escalation in three different concentration arms [22]. Figure 4 presents the network of the systematic review. Modalities compared in the meta-analysis and outcomes under each comparison are reported below.

#### 3.2.1. Modalities: Prolotherapy Versus Placebo

##### Outcome: Visual Analogue Pain

**Figure 5:**
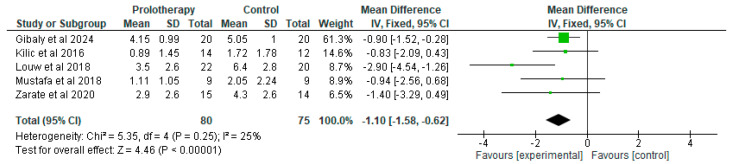
Forest plot of comparison [16,20,22,23,24]: 1 Prolotherapy versus Placebo; outcome: 1.1 VAS for pain.

Five studies examined the VAS for pain outcome under theprolotherapy versus a placebo comparison [16,20,22,23,24]. Refai et al. [21] reported the outcome as percentage changes in an ordinal variable; it was not feasible to convert this to a single score. One only used deep dry needling to simulate a placebo effect [16] while the remainder used normal saline instead of dextrose solution [20,22,23,24]. The visual analogue pain score was reported by all of them in a timeframe between three and five months. Three studies reported statistically non-significant differences [20,22,23], while two studies reported statistically significant differences between treatment modalities [16,24]. The trend was in the same direction for all studies, including the study by Refai which was not pooled, resulting in low heterogeneity (Figure 5).

##### Outcome: Maximal Incisor Opening

**Figure 6:**
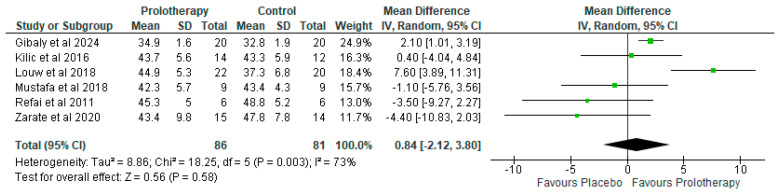
Forest plot of comparison [16,20,21,22,23,24]: 1 Prolotherapy versus Placebo; outcome: 1.3 maximal incisor opening.

Six studies recorded maximal incisor opening in a timeframe between three and five months. Two reached statistical significance favouring prolotherapy, [16,24], one was in the middle not favouring any treatment [20], and three favoured placebo, although not statistically significantly (Figure 6) [21,22,23]. Heterogeneity was high, and therefore the random effects model was used. This limitation is further analyzed in the discussion.

##### Outcome: Subluxation/Jaw Mobility

**Figure 7:**
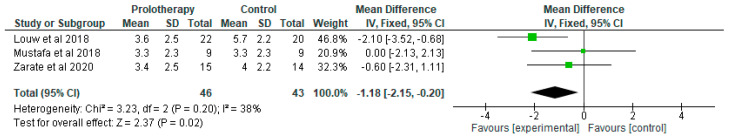
Forest plot of comparison [22,23,24]: 1 Prolotherapy versus Placebo; outcome: 1.2 Subluxation/Mobility.

Three studies reported on subluxation of the mandible. Mustafa et al. did not report on a quantitative score; instead, they provided the percentage of patients complaining about subluxation prior to and after the intervention. The rate was similar for both their groups (prior 77% and after 33%) which was quantified and added to a 10-scale and the forest plot. Despite this fact and that one of two other studies did not achieve statistical significance on their own [23,24], the overall effect reached significance with low–moderate heterogeneity (Figure 7).

#### 3.2.2. Modalities: Prolotherapy Versus Autologous Blood Products

##### Outcome: Visual Analogue Pain

**Figure 8:**
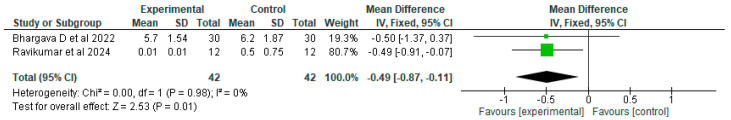
Forest plot of comparison [18,26]: 2 Prolotherapy versus autologus blood products; outcome: 2.1 VAS for pain.

Two studies reported on the active comparator of blood product injections. Bhargava et al. [18] used autologous blood injections as active comparators, while Ravikumar et al. used [26] autologous conditioned serum (whole blood incubated for 3 h at 37 °C, then centrifuged for 5 min at 4000 RPM, then serum supernatant was obtained and injected). The latest study [26] reported a significant result, which was on the same magnitude as the Bhargava et al. [18] study (Figure 8). This result is further analyzed in the discussion.

#### 3.2.3. Modalities: Prolotherapy Versus Botulinum Toxin

Kilic et al., in a subsequent trial, examined the outcome of Botulinum Toxin (BTX-A) use against prolotherapy. They reported the mean frequency of their primary outcome, locking episodes to be 3.10 ± 1.22 years in the BTX-A group and 2.50 ± 1.23 in the prolotherapy group. The result did not reach significance.

#### 3.2.4. Modalities: Ultrasound-Assisted Prolotherapy

Alhaj Kheder et al. [17] recently published results from an RCT that examined the effect of ultrasound guidance in the application of prolotherapy. TMJ VAS for pain was the main outcome. They reported that 2 months after the intervention, VAS for pain was 2.09 ± 2.02 in the U/S-assisted group as opposed to 2.18 ± 1.83 in the non-U/S-assisted group. It is worth noting that their control baseline scores were lower than in the experimental arm, and the randomization process was not explicitly reported.

#### 3.2.5. Modalities: Prolotherapy Versus Occlusal Splints

Priyadarshini et al. examined the efficacy of prolotherapy using occlusal splints as an active comparator in a randomized controlled setting of 34 patients with temporomandibular joint internal derangement classed as Wilkes stages II or III [27]. Both groups improved; however, prolotherapy group patients demonstrated statistically significant improvement in pain (*p* < 0.001), mouth opening (*p* = 0.032), and clicking (*p* < 0.001) when compared to occlusal splint controls. No significant difference in the deviation was observed between the groups after 1 year (*p* = 0.862).

#### 3.2.6. Modalities: Different Dextrose Concentrations

The majority of studies in this systematic review used a dextrose injection concentrate ranging between 8 and 25%. More specifically in increasing order 8%(+Bupivacaine) [18], 10% [17], 12% [19,20], 12.5% [26,27], 13.5% [21], 20% [24] and 25% [16,25] were used, while 12.5% to 25% variable concentrations were used in a single study [23]. Mustafa et al. [22] designed their study to examine the possible difference between 10%, 20%, and 30% dextrose content when added 1: 1 to lidocaine 2% solution, resulting in 5%, 10%, and 15% injected solution final dextrose contents. For better comparability, the medium group was used for meta-analyses purposes in the present study, as suggested above. They randomly allocated nine patients to each group. Post-intervention VAS for pain were as follows. For the control: 1.77 ± 1.64; for the 10%D group: 0.7 ± 0.67; for the 20%D group: 0.55 ± 0.72; and for the 30%D group: 0.88 ± 0.6 [22]. They were not able to determine any statistically significant differences throughout the study intervals between these groups.

## 4. Discussion

The aim of the present study was to identify qualitatively and quantitatively synthesized results from existing randomized controlled trials examining prolotherapy as a treatment modality for TMDs. We were able to identify studies comparing prolotherapy to placebo but also a few studies comparing prolotherapy to other treatments. The results have been presented and are discussed immediately below.

There was low heterogeneity in this meta-analysis, with regard to the beneficial effect of prolotherapy in reducing patient-perceived pain (Figure 5). The mechanisms underlying pain reduction are not fully understood. The pathophysiology of the therapeutic effect of prolotherapy is not very accurately established [28,29]. The possible proliferation of cells, namely macrophages and lymphocytes, by the employment and cooperation of locally existing fibroblasts, could augment the inflammatory cascade, possibly leading to improved healing [30]. Furthermore, dextrose has been proposed to have a beneficial effect on cartilage tissue [31]. It is worth noting that dextrose is a precursor for the synthesis of essential blocks of cartilage tissue, namely glycosaminoglycans, glycoproteins, and glycolipids [31]. What is more, dextrose has been demonstrated to upregulate the expression of aggrecan, a 2316-amino-acid-long glycoprotein in chondrocytic cell lineages. Aggrecan could alter the osmotic properties of the intracellular matrix, possibly allowing for the better growth and function of chondrocytes and fibroblasts [32]. Apart from cartilage tissue, dextrose may also be beneficial for neural tissue [33]. Burdakov et al. [34] reported the glucose-mediated inhibition of orexin/hypocretin neurons, which promote wakefulness (their loss causes narcolepsy) and also regulate metabolism and reward. The underlying mechanism might be related to the opening of potassium channels, thereby hyperpolarizing the nerve cells and lessening signal transmission [34]. Similarly, glucose solutions have been reported to block pain by other, not very well-described mechanisms. It has been suggested that subcutaneous prolotherapy injections of hypertonic glucose and 0.1% lignocaine might induce apoptosis of proliferating peptidergic noceffectors and neovessels by reducing VEGF levels and restoring “effective repair” processes, with the final outcome being the reduction in pain [35]. Transient Receptor Potential (TRP) channels have emerged as potential drug targets for the treatment of osteoarthritis, rheumatoid arthritis, and gout. Their role is likely facilitated by allowing sodium and calcium ions to enter the cell [36]. The permeability of those channels has been suggested to be altered by glucose in the extracellular environment [8]. In the clinical setting, such a mechanism is rendered plausible via the established application and favourable effect of prolotherapy in epidural injections for the treatment of chronic low back pain [13,37,38,39,40], intra-articular injections for knee pain [11,12,41,42], carpal tunnel syndrome [32,43], Achilles tendonitis [43,44,45], and recently rib fractures [46].

With regard to maximal incisor opening (MIO), Refai et al. [21] and Mustafa et al. [22] used both intra-articular and extra-articular injections. Thus, their worse MIO (Figure 6) may be attributed to the patients in those two trials having painful subluxation or dislocation of the TMJ. Thus, the reduction in MIO was an anticipated favourable outcome, aiming to improve the temporomandibular complex stability. Extra-articular injections have been previously reported in case series studies to help reduce jaw mobility [47,48]. Patients included in the other three trials (Figure 6) had painful clicking TMJ, without subluxation or dislocation. Therefore, the high heterogeneity recorded in this outcome is owing to the different pathologies being included. This high heterogeneity is actually a verification of the proper conduction of the analyses. It has been suggested that extra-articular injections, with trauma owing to numerous injection attempts, combined with the proliferative effects of dextrose, could augment the inflammatory response [30,32], ultimately causing fibrosis, which can be a favourable effect leading to TMJ capsule solidification.

TMJ subluxation/hypermobility was examined by the three included RCTs (Figure 7). The divergence in the MIO synthesis of these studies and has already been discussed immediately above. This comparison includes the Mustafa et al. study, [22], which is completely balanced between arms with regard to this outcome. Louw et al. [24] reported a statistically significant difference, while the results by Zarate et al. [23] are similar but not significant statistically. This outcome will need to be further elucidated, while also keeping in mind the discussion above regarding the pathophysiology of joint stabilization following prolotherapy. The standardization of the diagnostic criteria of included patients with subluxation/hypermobility is crucial for future syntheses to be productive.

Autologous blood products were used in the two studies, each one having exactly the same effect size (Figure 8). This zero heterogeneity is indicative of an advantage of prolotherapy when compared to autologous blood active comparators. Further to the study by Ravikumar et al. [26] and because platelet-rich fibrin protocols are currently well established and thought to be superior to platelet-rich plasma nowadays [49,50], future RCTs might prudently include platelet-rich plasma-active comparator arms against prolotherapy. Such studies need to report RCF (G-force) for centrifuge protocols as RPM with an unknown centrifuge rotor diameter does not allow for reproducibility.

Ultrasound-assisted prolotherapy showed marginally but not statistically better results as opposed to free-hand injection [17]. It is important to note that the single study from which this result is derived, although randomized, did not report the random allocation method (Figure 2). Notably, their control baseline scores were lower than in the experimental arm; therefore, we advocate that future research is required to confirm this negative result, especially since ultrasound had been reported to be a useful adjunct in the prolotherapy of other morbidities like cluneal neuropathy [38], tendinopathies [43], lumbar stenosis [13], and rib fractures [46]. Of course, the limited size and cutaneous proximity of the TMJ might allow experienced maxillofacial surgeons to inject equally efficiently with the ultrasound guidance, which would be more useful, i.e., in the rib cage.

The use of dental occlusal splints as an active comparator has been reported in a single study of TMD patients with internal derangement [26]. The results were clearly superior in the prolotherapy arm (for pain, mouth opening, and clicking), with the exemption of deviation. Since occlusal splints are considered a good therapeutic modality for TMDs [51], this supremacy of prolotherapy to splints is quite useful for clinical practice and needs to be further verified.

Prolotherapy dextrose concentration was variable among studies included in this systematic review, ranging from 8% to 25% in the final injectable solution. Mustafa et al. [22] were not able to detect any meaningful differences. We advocate that according to our analyses, any concentration between 10 and 20% would be sufficient for the beneficial effects of prolotherapy to manifest.

Limitations of the present study include a small number of available RCTs, with few patients in each one of them. Another limitation is that owing to the different methods of reporting outcomes, it was not feasible to include some studies in the quantitative syntheses. We also had to calculate the SD from the IQR for one study [26], while in another one, we had to convert a percentage to a scale, although with exactly identical performance of both prolotherapy and control studied groups, [22] thereby actually strengthening the pooled result. Not all included studies used the same diagnostic RDC/TMD criteria to classify included patients. Maximal incisor opening pooled comparison comprises both studies with TMJ pain patients, as well as studies with jaw hypermobility, clicking, or even subluxation symptoms. This was noted in the analyses and the random effects model was employed to compensate for this heterogeneity. Finally, despite the favourable quantified outcome in pain which is statistically significant (Figure 5) and uniformly reported by included studies, the follow-up time rarely exceeded 6 months, with the median being 3 months; therefore, future studies may need to address the long-term efficacy of prolotherapy in VAS for pain outcomes.

## 5. Conclusions

Prolotherapy is an emerging modality used to treat joint disorders [11,12,13,32,35,37,38,39,40,41,42,43,44,45,46]. In this regard, the present review qualitatively and quantitatively synthesized the currently available evidence about the application of prolotherapy in the temporomandibular joint. To the best of our knowledge, this work is more comprehensive than previously published systematic reviews on the topic [7,8,52], having the largest number of studies pooled under each outcome. The robust methodology and use of the random effects model to compensate for heterogeneity where applicable were employed. Future high-quality RCTs are needed, which would employ the study design propositions we make in the present discussion. Prolotherapy is clinically efficient in reducing TMD-related pain; this result is unlikely to be changed in future syntheses of new studies. The standardization of the diagnostic criteria for included patients would allow for future meta-analyses to obtain more robust results. Also, reporting treatment costs could allow for quantitative syntheses of the cost-effectiveness of prolotherapy.

## Figures and Tables

**Table 1:** Characteristics of included studies.

Author/Year	Type of Study	Age		Gender		Number of Patients		Type of Intervention
		SG	CG	SG	CG	SG	CG	
Gibaly et al., 2024 [16]	RCT	31.70 ± 7.81	31.85 ± 6.88	8 M (40%) 12 F (60%)	7 M (35%) 13 F (65%)	20	20	**S:** Four sessions of intra-articular and masseteric TP insertion of 1.5 mL of 12.5% dextrose solution in a fast in and fast out technique aided by a 25 gauge needle and 3.8 cm length with a time interval of 15 days between each session. **C:** Four sessions of intra-articular and masseteric DDN insertion in a fast in and fast out technique aided by a 25 gauge needle and 3.8 cm length with a time interval of 15 days between each session.
Alhaj Kheder et al., 2024 [17]	RCT	**u/s guided group** 24.3 ± 6.9	**Non u/s guided** 27.3 ± 7.4 years	**n = 22(u/s + non u/s)** 5 M 17 F	**n = 22(u/s + non u/s)** 5 M 17 F	**u/s** 11	**Non u/s** 11	**Non guided**: The injection was carried out according to the Hemwall–Hackett technique at three target points: (1) the posterior part of the joint space, (2) the anterior disc attachment, and (3) the most rigid point of the masseter. **U/S guided:** Two target points: (1) first injection intra-capsular and (2) second injection intramuscular (masseter muscle).
Bhargava D et al., 2022 [18]	RCT	29.2 ± 8.46	29.2 ± 8.46	14 M (46.6%) 16 F (53.33%)	18 M (60%) 12 F (40%)	30	30	**SG:** Here, 1 mL of heavy bupivacaine-dextrose was injected into the superior joint space and 2 mL in peri-articular and retro-discal region. **CG:** Here, 1 mL of the blood was deposited in the superior joint space via inflow needle and 2 mL in the peri-capsular and retro-discal region.
Kilic et al., 2022 [19]	RCT	**BTX-A GROUP:** 25.64 ± 7.35	**PROLOTHERAPY GROUP:** 32.36 ± 13.45	**BTX-A** 2 M (18.2%) 9 F (81.8%)	**PROLOTHERAPY** 4 M (29%) 10 F (71%)	**BTX-A** 11	**PROLOTHERAPY** 14	**BTX-A:** One session of BTX-A injection into lateral pterygoid muscles. **Prolotherarpy group:** Three sessions of intra- and peri-articular DP injections and a 1 mL injection of dextrose solution (2 mL 30% dextrose + 2 mL saline + 1 mL 2% articaine or mepivacaine) per injection area (posterior disc attachment, superior joint space, superior and inferior capsular attachments, and stylomandibular ligament) three times, each a month apart.
Kilic et al., 2016 [20]	RCT	32.36 ± 13.45	29.00 ± 9.24	4 M, 10 F	3 M, 9 F	14	12	**S:** Three injections of a dextrose solution (2 mL 30% dextrose, 2 mL saline, and 1 mL 2% articaine or mepivacaine), each a month apart. **C:** Three injections of a placebo solution (4 mL saline and 1 mL 2% articaine or mepivacaine) on the same schedule.
Refai et al., 2011 [21]	RCT	23 ± 2.75	29.83 ± 5.91	6 F	2 M, 4 F	6	6	**S:** Four injections of a dextrose solution (2 mL of 10% dextrose and 1 mL of 2% mepivacaine), each 6 weeks apart. **C:** Four injections of a placebo solution (2 mL of saline solution and 1 mL of 2% mepivacaine) on the same schedule.
Mustafa et al., 2018 [22]	RCT	**10%D:** 23.6 ± 7.32 **20%D:** 27.1 ± 7.67 **30% D:** 24.5 ± 4.21	25.3 ± 7.43	**10%D** 3 M, 7 F **20%D** 1 M, 8 F **30%D** 3 M, 6 F	4 M, 5 F	**10%D:** 10 **20%D:** 9 **30%D:** 9	9	**S:** Four injections of dextrose solution (3 mL solution of 1.5 mL solution of 10%, 20%, and 30% dextrose and 1.5 mL of 2% lidocaine), each a month apart. **C:** Four injections of placebo solution (a 3 mL solution of 1.5 mL of 0.9% saline solution and 1.5 mL of 2% lidocaine) on the same schedule.
Zarate et al., 2020 [23]	RCT	44.19 ± 15.1	50.1 ± 18.0	2 M (13%) 13 F (87%)	2 M (14%) 12 F (86%)	15	14	**S:** Three injections of 1 mL solution of 20% dextrose and 0.2% lidocaine. Each injection 4 weeks apart. **C:** Three injections of 1 mL of 0.2% lidocaine in sterile water.
Louw et al., 2018 [24]	RCT	44 ± 14.1	50 ± 13.4	8 tm joints M 22 tm joints F	1 TMJ M 23 tmj joints F	22 patients No of tmjs: 30	20 patients No of tmjs: 24	**S:** Three intra-articular injections of 1 mL solution of 20% dextrose and 0.2% lidocaine, each one month apart. **C:** Three intra-articular injections of 1 mL solution of 0.2% lidocaine in sterile water.
Priyadarshini et al., 2020 [27]	RCT	31.76	28.35	7 M (41.2%) 10 F (58.8%)	5 M (29.4%) 12 F (70.6%)	17	17	**S:** Four injections of solution (dextrose 50% (0.75 mL), lidocaine 2% with adrenaline (1.5 mL), and bacteriostatic water (0.75 mL). First injection on day 1; second injection 2 weeks after the first injection; third injection 4 weeks after the second injection; and fourth injection 6 weeks after the third injection. **C:** Anterior bite planes were given to patients in the control group, which produced a posterior open bite of 2 mm. Patients were advised to wear splints for 12 h per day for up to 3 months.
Haggag et al., 2022 [25]	RCT	22.7	23.9	15 F (100%)	15 F (100%)	15	15	**S:** Intra-articular injection of 2 mL solution of 25% dextrose after auriculotemporal nerve block. Up to a maximum of four injections at weekly intervals, according to the complete satisfaction of the patient. **C:** Intra-articular injection of 2 mL solution of normal saline after auriculotemporal nerve block. Up to a maximum of four injections at weekly intervals, according to the complete satisfaction of the patient.
Ravicumar et al., 2024 [26]	RCT	23.25 ± 7.04	28.75 ± 8.45	1 Μ (8.3) 11 F (91.7)	4 M (33.3) 8 F (66.7)	12	12	**S:** Four injections of 3 mL of autologous blood serum in three different sites: posterior joint space, anterior disc attachment to the lateral pterygoid muscle, and masseter attachment, with 1 mL in each site. First day, second week, fourth week, and sixth week. **C:** Four injections of 3 mL solution (50% dextrose (0.75 mL), 1.5 mL lidocaine 0.2% with adrenaline, and 0.75 mL of bacteriostatic water). The same technique as mention above. On the same schedule.

S: study group; C: control group; TMJ: temporomandibular joint; DDN: Deep Dry Needling; TP: trigger points; S: study; C: control; M: male; F: female; RCT: Randomized Controlled Trial; tm: temporomandibular; D: dextrose; BTX-A: Botulinum Toxin A; TMJ: temporomandibular joint; U/S: ultrasound.

**Table 2:** Outcome assessment by included studies.

Author/Year	Maximum Inter-Incisal Mouth Opening (mm)		Frequency of Luxation/TMJ Dysfunction		TMJ Pain VAS Measurement		Clicking/ Presence/Absence (P/A)	
	Pre-Op	Post-Op	Pre-Op	Post-Op	Pre-Op	Post-Op	Pre-Op P/A	Post-Op P/A
Gibaly et al., 2024 [16] Evaluation of the effect **of dextrose prolotherapy versus deep dry needling therapy** for the treatment of temporomandibular joint anterior disc displacement with reduction: (a randomized controlled trial)	**S:** 29.40 ± 1.90 **C:** 30.15 ± 1.93	**1 month** **S:** 32.15 ± 1.18 **C:** 31.50 ± 2.35 **2 months** **S:** 34.60 ± 1.47 **C:** 32.55 ± 2.09 **5 months** **S:** 34.95 ± 1.61 **C:** 32.80 ± 1.96 **8 months** **S:** 35.90 ± 1.48 **C:** 32.85 ± 2.13	NR	NR	**S:** 7.70 ± 0.86 **C:** 7.75 ± 0.85	**1 month** **S:** 6.50 ± 1.24 **C:** 5.55 ± 1.05 **2 months** **S:** 4.95 ± 1.15 **C:** 5.15 ± 0.93 **5 months** **S:** 4.15 ± 0.99 **C:** 5.05 ± 1.00 **8 months** **S:** 4.05 ± 0.76 **C:** 5.20 ± 1.01	**S:** 20/0 100%/0% **C:** 20/0 100%/0%	**1 month** **S:** 17/3 (85%/15%) **C:** 19/1 (95%/5%) **2 months** **S:** 14/6 (70%/30%) **C:** 17/3 (85%/15%) **5 months** **S:** 13/7 (65%/35%) **C:** 18/2 (90%/10%) **8 months** **S:** 15/5 (75%/25%) **C:** 17/3 (85%/15%)
Alhaj Kheder et al., 2024 [17] **Ultrasound-Guided Vs. Non Guided** Prolotherapy for Internal Derangement of Temporomandibular Joint. A Randomized Clinical Trial	NR		NR		**u/s:** 4.27 ± 1.62 **Nu/s:** 3.91 ± 1.04	**4 weeks** **u/s**: 3.18 ± 2.23 **Nu/s**: 3.36 ± 1.86 **2 months** **u/s:** 2.09 ± 2.02 **Nu/s:** 2.18 ± 1.83 **6 months** **u/s:** 0.7 ± 1.25 **Nu/s:** 1 ± 0.53	NR	
Bhargava D et al., 2022 [18] A Comparative Preliminary Randomized Clinical Study to Evaluate **Heavy Bupivacaine Dextrose Prolotherapy (HDP) and Autologous Blood Injection (ABI)** for Symptomatic Temporomandibular Joint Hypermobility Disorder	**S:** 43.27 ± 7.49 **C:** 42.86 ± 6.89	**6 months** **SG**: 38.52 ± 5.41 **CG**: 39 ± 5.84 **12 months** **SG:** 37.87 ± 1.98 **CG**: 38.42 ± 2.57	NR		**S:** 8.4 ± **C:** 8.9 ±	**6 months** **SG**: 5.7 ± 1.54 **CG**: 6.2 ± 1.87 **12 months** **SG**: 4 ± 1.2 **CG**: 4.7 ± 1.17	NR	
Kilic et al., 2022 [19] **Botulinum Toxin Versus Dextrose Prolotherapy**: Which is More Effective for Temporomandibular Joint Subluxation—A Randomized Clinical Trial	NR	NR	**Frequency of locking episodes** **(5-grade scale)** **BTX-A** 3.10 ± 1.22 **Prolotherapy** 2.50 ± 1.23	**WILCOXON SIGNED RANK TEST** **Follow-up** Intragroup comparisons of the primary outcome variable showed that the frequency of locking episodes **decreased significantly in both groups**.	**Patient satisfaction** **(5-grade level)** **MANN**–**WHITNEY U TEST** **81.8 percent** of patients in the **BTX-A** group and **71.4 percent in the prolotherapy group** reported their high pleasures (good plus excellent), respectively. Patient satisfaction showed **no statistically significant difference** between the groups (*p* > 0.05).	**Patient satisfaction** **(5 grade level)** **MANN**–**WHITNEY U TEST** **81.8 percent** of patients in the **BTX-A** group and **71.4 percent in the prolotherapy group** reported their high pleasures (good plus excellent), respectively. Patient satisfaction showed **no statistically significant difference** between the groups (*p* > 0.05).	NR	
Kilic et al., 2016 [20] **Is dextrose prolotherapy superior to placebo** for the treatment of temporomandibular joint hypermobility? A randomized clinical trial	**S:** 46.14 ± 6.89 **C:** 46.33 ± 3.47	**S:** 43.29 ± 5.92 **C:** 43.67 ± 5.65	NR	NR	**S:** 4.30 ± 2.57 **C:** 5.39 ± 2.09	**S:** 0.89 ± 1.45 **C:** 1.72 ± 1.58	NR	
Refai et al., 2011 [21] The Efficacy of **Dextrose Prolotherapy** for Temporomandibular Joint Hypermobility: A Preliminary Prospective, Randomized, Double-Blind, **Placebo**-Controlled Clinical Trial	**S:** 50.3 ± 4.3 **C:** 49.7 ± 4.9	**S:** 43.3 ± 4.5 **C:** 49.7 ± 4.8	NR	Number of episodes from last injection to follow up **S:** 0.2 ± 0.4 **C:** 0.3 ± 0.8	**Measured on verbal scale. No numerical data available**. Comparison between the two groups showed **insignificant statistical difference in pain intensity** throughout the study intervals.	**Measured on verbal scale. No numerical data available.** Comparison between the two groups showed **insignificant statistical difference in pain intensity** throughout the study intervals.	NR	
Mustafa et al., 2018 [22] Evaluation of the Efficacy **of Different Concentrations of Dextrose Prolotherapy** in Temporomandibular Joint Hypermobility Treatment	**10%D:** 54.3 ± 5.92 **20%D** 52.11 ± 6.9 **30%D** 54 ± 7.41 **C:** 52.33 ± 6.33	**10%D:** 39.4 ± 4.19 **20%D** 41.22 ± 5.4 **30%D:** 39.44 ± 4.55 **C**: 43.44 ± 4.24	Number of cases of open lock: **10%D**: 9 **20%D**: 4 **30%D**: 6 **C:** 6	Number of cases of open lock: **10%D:** 0 **20%D:** 0 **30%D:** 0 **C:** 0	**10%D:** 5.25 ± 2.84 **20%D** 5.66 ± 1.95 **30%D:** 5.33 ± 2.29 **C:** 4.38 ± 3.14	**10%D:** 0.7 ± 0.67 **20%D:** 0.55 ± 0.72 **30%D:** 0.88 ± 0.6 **C:** 1.77 ± 1.64	NR	
Zarate et al., 2020 [23] **Dextrose Prolotherapy Versus Lidocaine Injection** for Temporomandibular Dysfunction: A Pragmatic Randomized Controlled Trial	**S:** 38.7 ± 10.6 **C:** 42.4 ± 9.27	**3 months** **S:** 43.4 ± 9.8 **C**: 47.8 ± 7.8	**TMJ dysfunction** **(NRS 0–10)** **S:** 7.4 ± 1.0 **C:** 7.1 ± 0.9	**TMJ dysfunction** (**NRS 0–10)** **1 month** **S:** 4.0 ± 2.7 **C:** 5.9 ± 1.5 **2 months** **S:** 3.9 ± 2.7 **C:** 4.6 ± 2.2 **3 months** **S:** 3.4 ± 2.5 **C:** 4.0 ± 2.2 **12 months** **S:** 2.0 ± 2.4 **C:** 4.4 ± 2.5	**S: 7.2** ± **(1.1)** **C: 7.2** ± 0.8	**1 month** **S:** 4.4 ± 2.4 **C:** 5.4 ± 2.1 **2 months** **S:** 4.4 ± 2.4 **C:** 4.6 ± 2.2 **3 months** **S:** 2.9 ± 2.6 **C:** 4.3 ± 2.6 **12 months** **S:** 2.4 ± 2.6 **C:** 4.6 ± 2.5	NR	
Louw et al., 2018 [24] Treatment of Temporomandibular Dysfunction with **Hypertonic Dextrose Injection** (Prolotherapy): A Randomized Controlled Trial with Long-term Partial Crossover	**S:** 43.4 ± 5.7 **C:** 39.0 ± 6.8	**Ghange scores** **0**–**3 months** **S:** 1.5 ± 4.1 **C**: −1.8 ± 5.1 **0**–**12 months** **S:** 1.3 ± 4.9 **C:** 3.1 ± 6.2	**S: 7.2** ± **1.1** **C: 6.7 ± 0.9**	**Ghange scores** **1 month** **S:** 1.5 ± 1.9 **C:** 0.2 ± 0.5 **2 months** **S:** 2.8 ± 2.7 **C:** 0.8 ± 1.3 **3 months** **S:** 3.5 ± 2.8 **C:** 1.0 ± 2.1 **12 months** **S:** 4.2 ± 2.9 **C:** 4.0 ± 2.7	**S:** 7.8 ± 1.2 **C:** 8.2 ± 1.2	**Ghange scores** **1 month** **S:** 2.2 ± 1.8 **C:** 0.9 ± 1.4 **2 months** **S:** 3.3 ± 2.9 **C:** 1.8 ± 2.3 **3 months** **S:** 4.3 ± 2.9 **C:** 1.8 ± 2.7 **12 months** **S:** 5.1 ± 3.0 **C:** 5.4 ± 2.8	NR	NR
Priyadarshini et al., 2020 [27] Evaluation of **prolotherapy** in comparison with **occlusal splints** in treating internal derangement of the temporomandibular joint—A randomized controlled trial	**S:** 36.0 ± 11.00 **C:** 33.88 ± 9.13	**1 month** **S:** 40.65 ± 8.2 **C:** 34.71 ± 8.40 **3 months** **S:** 41.18 ± 8.00 **C:** 34.65 ± 8.38 **6 months** **S:** 41.35 ± 7.96 **C:** 34.82 ± 8.34 **12 months** **S:** 41.29 ± 7.96 **C:** 35.06 ± 8.22	NR	NR	**S: 5.76** ± 1.95 **C: 5.35** ± 1.93	**1 month** **S:** 0.59 ± 0.50 **C:** 3.47 ± 2.03 **3 months** **S:** 0.59 ± 0.50 **C:** 3.41 ± 1.93 **6 months** **S:** 0.47 ± 0.51 **C:** 3.41 ± 1.87 **12 months** **S:** 0.47 ± 0.51 **C:** 3.29 ± 1.82	**Clicking** **(Helkimo clinical dysfunction index)** Pre-op **S:** 2.24 ± 0.75 **C:** 2.82 ± 0.39	**Clicking** **(Helkimo clinical dysfunction index)** **1 month** **S:** 1.82 ± 0.63 **C:** 1.76 ± 0.66 **3 months** **S:** 1.53 ± 0.8 **C:** 1.76 ± 0.66 **6 months** **S:** 0.94 ± 0.74 **C:** 1.82 ± 0.72 **12 months** **S:** 0.82 ± 0.63 **C:** 1.82 ± 0.63
Haggag et al., 2022 [25] **Dextrose prolotherapy** for pain and dysfunction of the TMJ reducible disc displacement: A randomized, double-blind clinical study	**Measured on chart. No other numerical data.** Comparison between the two groups showed **significant improvement in MIO** (study group vs. control group) throughout the study intervals.	**Measured on chart. No other numerical data**. Comparison between the two groups showed **significant improvement in MIO** (study group vs. control group) throughout the study intervals.	NR	NR	**Measured on chart. No other numerical data**. Comparison between the two groups showed **significant improvement in pain intensity for dextrose group** after the 4th week of first injection.	**Measured on chart. No other numerical data** Comparison between the two groups showed **significant improvement in pain intensity for dextrose group** after the 4th week of first injection.	**Measured on chart. No other numerical data.** Comparison between the two groups showed insignificant statistical difference in pain in joint sounds throughout the study intervals.	**Measured on chart. No other numerical data.** Comparison between the two groups showed insignificant statistical difference in pain in joint sounds throughout the study intervals.
Ravicumar et al., 2024 [26] Evaluation of the efficacy of **autologous conditioned serum versus dextrose prolotherapy** in internal derangement of the TMJ—A pilot study	**S:** 29.33 ± 5.26 **C:** 30.25 ± 6.55	**2 weeks** **S:** 36.75 ± 3.95 **C:** 36.33 ± 4.53 **1 month** **S:** 39.17 ± 2.72 **C:** 37.17 ± 4.64 **2 months** **S:** 43.00 ± 3.83 **C:** 37.50 ± 4.46	NR	NR	**Mann–Whitney U test** **S:** 6.5 (6.0–8.5) **C:** 7.0 (5.5–8.0)	**Mann–Whitney U test** **2 weeks** **S:** 4.0 (1.5 4.0) **C:** 1.0 (0 1) **1 month** **S:** 1.0 (0 2.5) **C:** 1.0 (0 1) **2 months** **S:** 0 (0 0) **C:** 0 (0.5 1)	**Clicking (Yes/No)** **S:** 8y/4n **C:** 12y/0n	**Clicking (Yes/No)** **2 weeks** **S:** 4y/8n **C:** 12y/0n **1 month** **S:** 2y/10n **C:** 5y/7n **2 months** **S:** 1y/11n **C:** 3y/9n

S, study group; C, control group; NR, not reported; VAS, visual analogue scale 10 cm measurement; u/s, ultrasound-guided; Nu/s, non ultrasound-guided; SG, study group; CG, control group; TMJ: temporomandibular joint; MIO: maximal interincisal opening; VAS: visual analogue scale; S: study; C: control; D: dextrose; BTX-A: Botulinum Toxin A; TMJ: temporomandibular joint.

## Data Availability

More data and results are available on demand.

## Supplementary Materials

The following supporting information can be downloaded at https://www.mdpi.com/article/10.3390/clinpract15030051/s1. Figure S1. Funnel plot of comparison: 1 Prolotherapy versus Placebo, outcome: 1.1 VAS Pain. Figure S2. Funnel plot of comparison: 1 Prolotherapy versus Placebo, outcome: 1.2 Subluxation/Mobility. Figure S3. Funnel plot of comparison: 1 Prolotherapy versus Placebo, outcome: 1.3 Maximal Incisor Opening. Figure S4. Funnel plot of comparison: 2 Prolotherapy versus Autologus blood products, outcome: 2.1 VAS Pain. Table S1. Database search strategy. Table S2. PICO Framework. Table S3. Inclusion—Exclusion Criteria.
