# Correction: Saramantos et al. Clinical Efficacy of Prolotherapy for Temporomandibular Joint Disorders: A Systematic Review and Meta-Analysis. *Clin. Pract.* 2025, *15*, 51

**DOI:** 10.3390/clinpract16020034

**Published:** 2026-02-03

**Authors:** Antonios Saramantos, Athanassios Kyrgidis, Gregorios Venetis, Georgios Hatziantoniou, Anestis Chrysostomidis, Chrysanthi Sardeli, Ioannis Tilaveridis

**Affiliations:** 1 Department of Oral & Maxillofacial Surgery, Aristotle University of Thessaloniki, Specialized Cancer Treatment and Reconstruction Center, General Hospital of Thessaloniki “George Papanikolaou”, 57010 Thessaloniki, Greece; saramantosant@gmail.com (A.S.); ghatziadoniou@gmail.com (G.H.); a.chrisostomidis@gmail.com (A.C.); jtilaver@yahoo.com (I.T.); 2 Laboratory of Oral & Maxillofacial Surgery, Dental School, Aristotle University of Thessaloniki, 54124 Thessaloniki, Greece; gvenetis@dent.auth.gr; 3 Laboratory of Clinical Pharmacology, Medical School, Aristotle University of Thessaloniki, 54124 Thessaloniki, Greece; sardeli@auth.gr

## 1. Errors in Figure

In the original publication [1], there was a mistake in Figure 1 as published. A few data points in the figure are incorrect. “Reports excluded: Not eligible (*n* = 20)” should be “Reports excluded: Not eligible (*n* = 18)”. The corrected Figure 1 appears below.

## 2. Text Correction

### Description of Results from Literature Search

In the original publication [1], there was an error in the first paragraph in the Results Section. “We identified 42 citations from all searches. After screening the titles and abstracts, we retrieved 30 full texts for further assessment. Of these, 20 were excluded for the following reasons: duplicate publications as conference abstracts (*n* = 2), trial without a control arm (*n* = 10), review (*n* = 4), and articles not related to the topic (*n* = 4)” should be changed to “We identified 42 citations from all searches. After screening the titles and abstracts, we retrieved 30 full texts for further assessment. Of these, 18 were excluded for the following reasons: trial without a control arm (*n* = 10), review (*n* = 4), and articles not related to the topic (*n* = 4)”.

The authors state that the scientific conclusions are unaffected. This correction was approved by the Academic Editor. The original publication has also been updated.

## Figures and Tables

**Figure 1:**
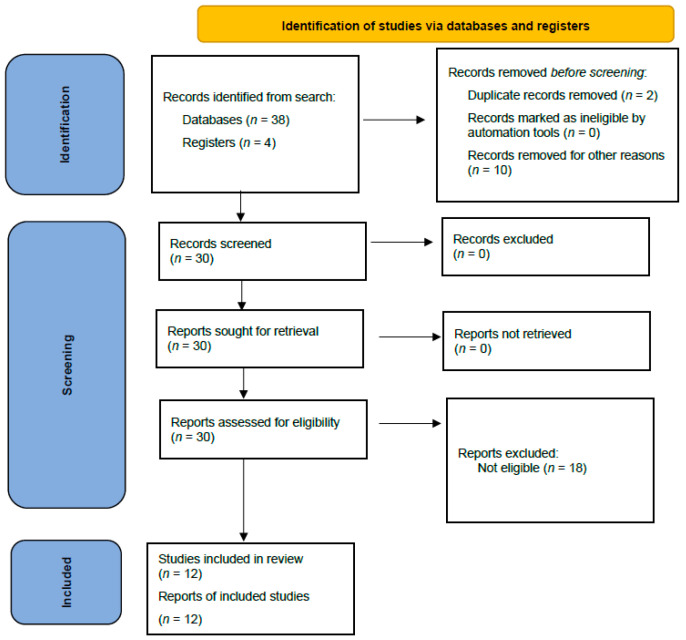
Flowchart of the systematic review.
