# Supplementary material for: Clinical Efficacy of Prolotherapy for Temporomandibular Joint Disorders: A Systematic Review and Meta-Analysis
Source: Clin Pract. 2025 Feb 27;15(3):51. doi: 10.3390/clinpract15030051 (PMC11941112; doi:10.3390/clinpract15030051)
Supplement: Supplementary file 1 [file clinpract-15-00051-s001.zip › clinpract-3442298-supplementary.pdf]

Table S1. Database search strategy.

Database	Keywords
MEDLINE	(("Tempormandibular"[MeSH Terms] OR "TMJ"[Title/Abstract] OR "Temporomandibular joint"[Title/Abstract] OR "temporo*"[Title/Abstract] OR "mandibular*"[Title/Abstract] OR " joint hypermobility "[Title/Abstract] OR " subluxation"[Title/Abstract]) AND ("prolotherapy"[MeSH Terms] OR "dextrose*"[Title/Abstract] OR "prolo*"[Title/Abstract] OR "dextrose prolotherapy "[Title/Abstract] OR "prolo*"[Title/Abstract]))
Scopus	('tempormandibular' OR TITLE-ABS ('mandibular') AND 'prolotherapy' OR TITLE-ABS ('prolotherapy') OR TITLE-ABS ('dextrose') AND PUBYEAR > 1990) AND NOT INDEX (medline)
Cochrane Library	ID Search<br>#1 MeSH descriptor: [temporomandibular] explode all trees<br>#2 prolotherapy<br>#3 dextrose<br>#4 MeSH descriptor: [dextrose] explode all trees 4987<br>#5 #1 or #2<br>#6 #1 or #3<br>#7 #3 or #4 or #5<br>#8 #6 and #7

Table S2. PICO Framework.

Population	Intervention	Comparator	Outcome
Patients presenting with temporomandibular disorders	Dextrose solution intraarticular infusion (prolotherapy)	Placebo<br>Autologous blood products<br>Botulinum Toxin<br>Occlusal Splints	VAS Pain<br>Maximal incisor opening (MIO)<br>Subluxation / Mobility

Table S3. Inclusion – Exclusion Criteria.

Inclusion Criteria	Exclusion Criteria
randomized controlled clinical studies that included patients with TMDs<br>prolotherapy had to be administered to at least one trial arm<br>the injection protocol ought to include an intra-articular injection, complemented or not by additional injections to the periarticular soft tissues.<br>placebo (i.e. normal saline injections) or active comparators (i.e.e blood injections) or other types of interventions (i.e. physiotherapy).	Non randomized trials<br>Cohort studies.<br>Case control studies.<br>Case series.<br>Case reports.<br>Articles not in English.<br>Animal studies.<br>Experimental studies.

Studies with multiple interventions were considered as long as they were uniform across all study groups such that the clean efficacy attributed to prolotherapy could be estimated.	

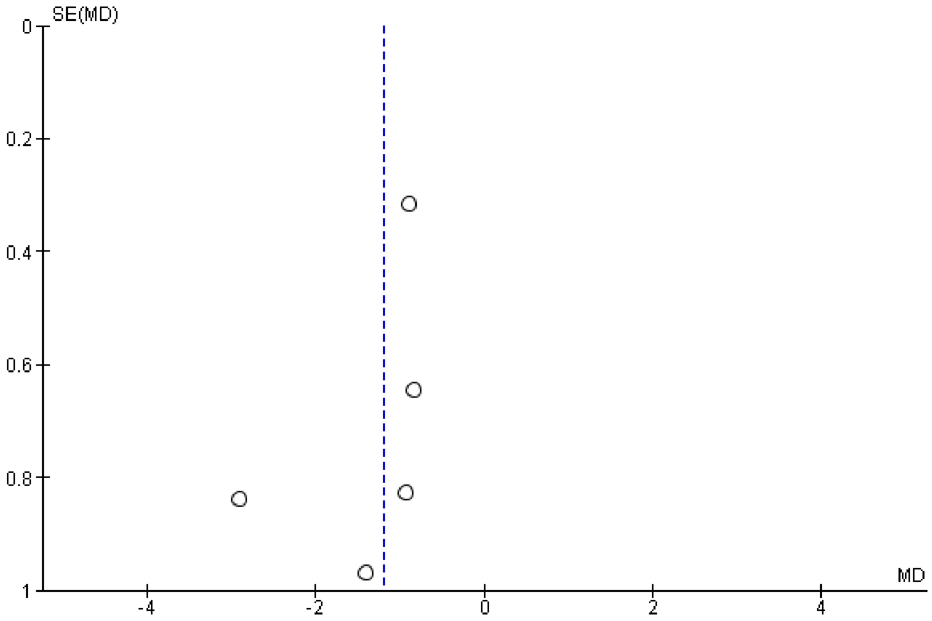

Figure S1. Funnel plot of comparison: 1 Prolotherapy versus Placebo, outcome: 1.1 VAS Pain.

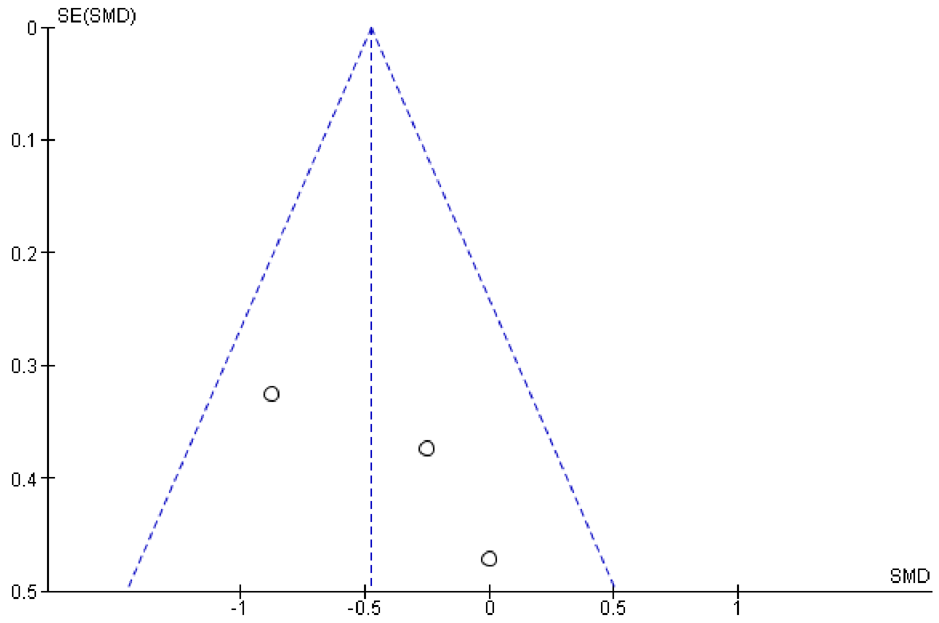

Figure S2. Funnel plot of comparison: 1 Prolotherapy versus Placebo, outcome: 1.2 Subluxation / Mobility.

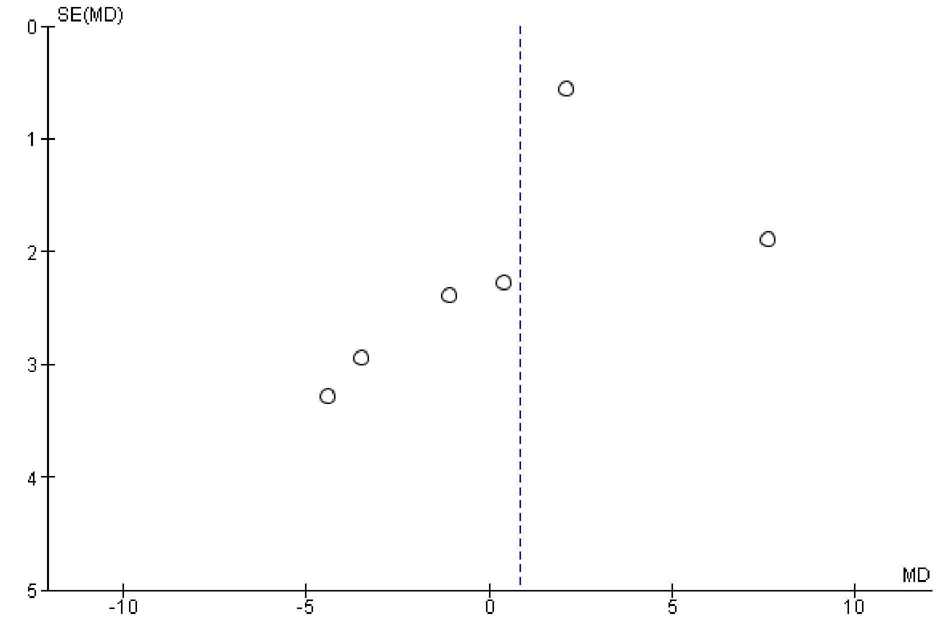

Figure S3. Funnel plot of comparison: 1 Prolotherapy versus Placebo, outcome: 1.3 Maximal Incisor Opening.

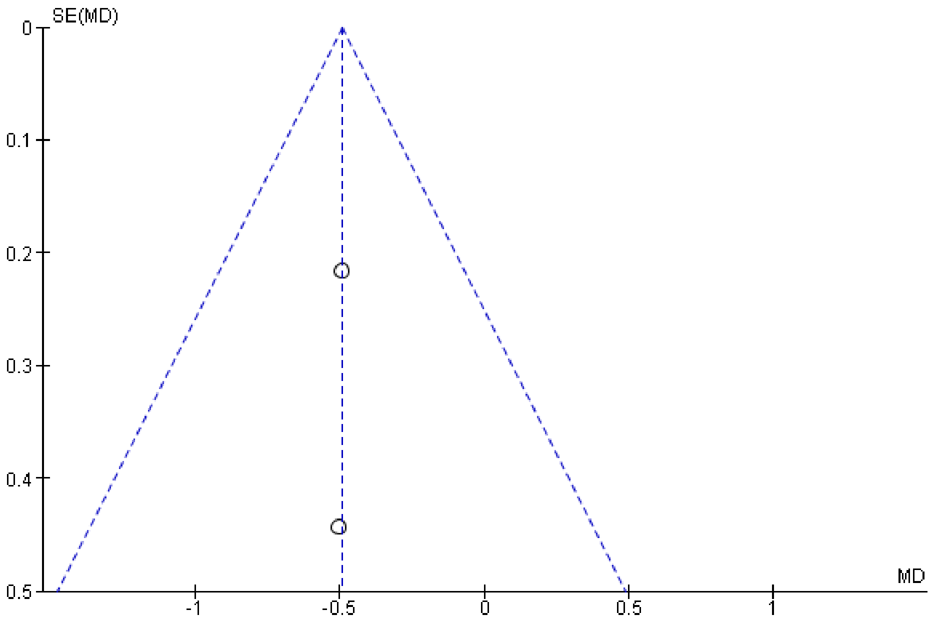

Figure S4. Funnel plot of comparison: 2 Prolotherapy versus Autologus blood products, outcome: 2.1 VAS Pain.
